# Focal hematopoietic hyperplasia of the rib—A form of pseudo-tumor. Case report and review of articles

**DOI:** 10.1016/j.ijscr.2019.12.011

**Published:** 2019-12-17

**Authors:** Reza Mollahosseini, Paniz Motaghi, Alireza Dastmalchi, Hanieh Zahm

**Affiliations:** a Iran University of Medical Sciences, Iran; b ShahidBehehsti University of Medical Sciences, Iran

**Keywords:** Case report, Chondral lesion, Rib mass, Hematopoietic mass, Hematopoietic hyperplasia

## Abstract

**Introduction:**

Focal hematopoietic hyperplasia (FHH) is an unusual benign lesion that can rarely involve the ribs. In general, FHHs is a tumor-like expansion of ribs due to the abnormal proliferation of bone marrow. FHHs are mostly found incidentally at radiologic studies performed for other reasons. They are characterized by gradually enlarging osteolytic masses that involve the rib.

**Presentation of case:**

Here, we present an unusual case of focal hematopoietic hyperplasia of the rib who arrived to our institute with a complaint of severing back pain at the level of T5-T6 vertebrae. The diagnosis of FHH has been made based on clinical and radiologic findings and confirmed by histopathologic evaluations. We also reviewed all previously reported cases of Focal Hematopoietic hyperplasia of the rib in the literature.

**Discussion:**

None of the previously reported cases, same as our patient, gave a history of trauma to the site involved or family history of malignancy. There was no association with hematological disorders or other malignancies that might have initiated FHH. Histologic evaluations of previously reported cases represented a hyper cellular bone marrow merging with the fatty marrow. The morphology and maturation of all hematopoietic cell lines were normal and there was no report of morphologically abnormal hematopoietic cell or malignant tumor cells.

**Conclusion:**

Physicians must consider the FHH among differential diagnosis of secondary rib lesions. Surgical resection is the treatment of choice; the risk of recurrence or metastasis is neglectable.

## Highlights

• Focal hematopoietic hyperplasia (FHH) is an unusual benign expansion of ribs due to the abnormal proliferation of bone marrow that can rarely involve the ribs.
• From the aspect of histopathology, FHH is a mix of hypercellular and fatty marrow. The cellularity was increased by approximately 50–95 %.
• FHH has no association with hematological disorders or other malignancies.
• Based on radiologic findings, fibrous dysplasia, aneurysmal bone cyst and plasmacytoma are among differential diagnosis of FHH.
• chondrosarcoma and some other bony lesions with the manifestation of hematopoietic hyperplasia must also be considered among differential diagnosis.

## 1 Introduction

Primary chest wall tumors are very rare conditions, comprising only 5%–10% of all bone neoplasm. Secondary chest wall tumors do not originate from ribs and their surrounding soft tissue. Metastatic disease is the most common process that involves chest wall. Chondrosarcoma, Ewing sarcoma, myeloma, and osteosarcoma are the most common primary malignant tumors. Yet, about 63 % of primary rib tumors are benign including osteochondroma, enchondroma, and fibrous dysplasia [1,2]. Benign and malignant tumors can be distinguished from each other based on radiologic findings and clinical features [1].

Focal hematopoietic hyperplasia (FHH) is an unusual benign lesion. Based on our review of articles they can very rarely involve the ribs. In general, FHHs is a tumor-like expansion of ribs due to the abnormal proliferation of bone marrow. So, they must be included in the differential diagnosis of primary rib lesions, especially, when assessing the adequacy of a specimen at the time of aspiration [3].

Previous cases of FHH were sporadic solitary lesions without any association with hematological disorders or other malignancies that might have initiated it [4].

FHHs are mostly found incidentally at radiologic studies performed for other reasons. They are characterized by gradually enlarging osteolytic masses that involve the rib [2].

Radiologically, this lesion appeared as an expansive and radiolucent formation and contained areas of increased density or calcification; the cortex was intact and no soft tissue extension was noted. Marrow changes were diffuse with a marked decrease in signal intensity of the spinal and pelvic bone marrow on T1-weighted MR imaging. Sparing of the epiphyses and absence of moderately increased signal intensity on T2-weighted sequences aid in differentiating reconverted red marrow from neoplasm [5].

Histologic evaluations of previously reported cases represented a hypercellular bone marrow merging with the fatty marrow. the morphology and maturation of all hematopoietic cell lines were normal and there was no report of morphologically abnormal hematopoietic cell or malignant tumor cells. For these reasons, they are considered a pseudo-tumor while radiologic and clinical findings are suggestive of a benign tumor [6,2,4].

Here, we present an unusual case of focal hematopoietic hyperplasia of the rib who arrived to our academic institution, Firouzgar General Hospital, Tehran, Iran with complaint of worsening back pain. The diagnosis has been made based on clinical and radiologic findings and confirmed by histopathologic evaluations. We also reviewed all previously reported cases of Focal Hematopoietic hyperplasia of the rib in the literature. This project has been reported in line with the SCARE criteria [7].

## 2 Case presentation

A twenty-two-year-old Iranian woman arrived at our institute with a complaint of severing back pain at the level of T5-T6 vertebrae since two months ago. There was no definite initiating cause and the pain was not associated with the activity. The pain did not radiate into any other part. She did not consume alcohol or illicit drug and had no history of underlying diseases.

Laboratory data were all within normal ranges except slightly elevated ESR.

**Fig. 1:**
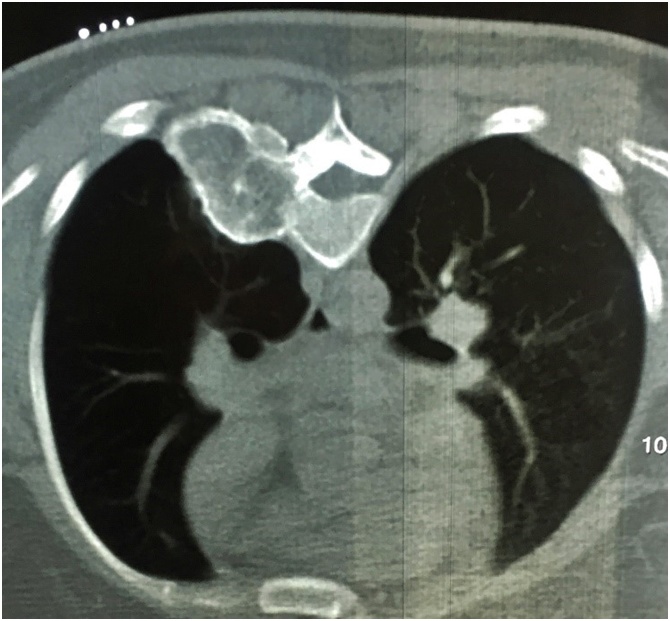
Chest Computed Tomography scan revealing the focal hematopoietic hyperplasia at the 6th rib.

The radiological study was performed. The Thorax spiral CT scanning without contrast injection was done and demonstrated an expansible bony lesion involving posterior arc of the right 6th rib, costovertebral joint and a portion of the T6 vertebrae. It was measured about 68*44 mm. In MRI, Right D5, D6, and D7 paravertebral mass were seen. The intervertebral foramen was also involved by the enhanced lesion (Fig. 1).

**Fig. 2:**
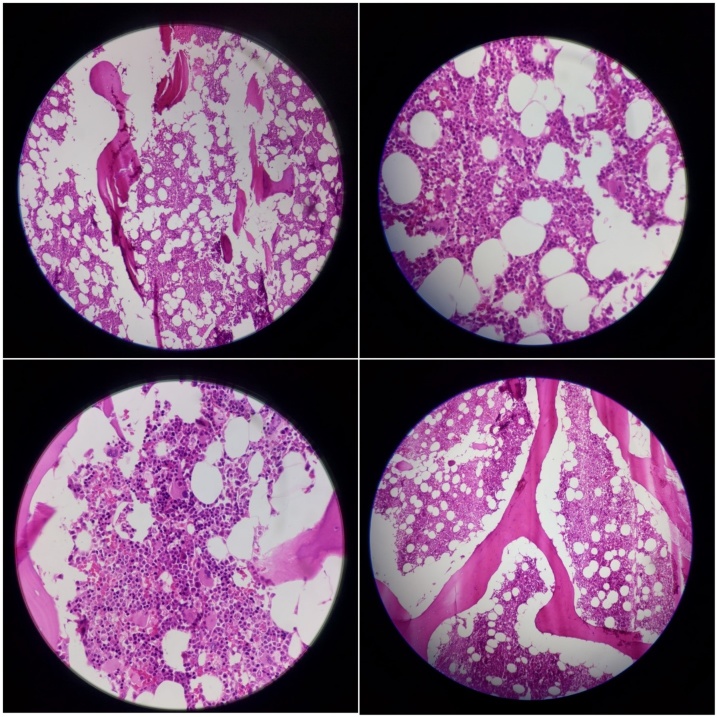
High-resolution images of the specimen which was suggestive of norm cellular bone marrow without malignant or atypical cells.

Then CT-guided biopsy and fine needle aspiration of the lesion was taken. A lobulated and irregular margin without cortical destruction was present. Macroscopic appearance of lesion was suggestive of benign osseous mass but extension into the body and right side of posterior elements of the adjacent vertebra was also seen. The specimen was sent for further evaluations. It contained multiple tiny pieces of brown soft tissue. The histopathologic finding was suggestive of norm cellular bone marrow. There were no malignant or atypical cell (Fig. 2).

Based on previous cases we decided to perform thoracostomy under general anesthesia by an expert spine surgeon. At the operating room, we performed resection of the proximal parts of right 6th rib and partial T5 and T6 corpectomy. During the surgery, we completely excised the lesion and a safe margin to avoid local recurrence. Intra-operatively frozen section was sent for rapid analysis to determine the safe margin from the lesion in surrounding parenchyma and minimize tissue damage. Although the mass had only involved the rib, and not the surrounding area, we gently removed reactive parts of the cortex for safety. Thus, intraoperative observation also helped in determining the margin of excision to decrease excessive tissue damage.

Then she was admitted at the Neurosurgery Intensive Care Unit for three days under our close observation. The post-operative period was uneventful. Wound healing occurred without infection or inflammatory response.

During one-year follow-up, she did not experience any abnormality. General physical exams were normal. Laboratory evaluations were all within standard ranges. However, we performed spiral chest and abdominopelvic CT scan; no lesion or any sign of remission was found.

Based on our review on literature, there were seven similar cases of Focal Hematopoietic hyperplasia reported in articles. Here we also compared our findings with their reports and discussed similar features.

## 3 Discussion

Focal hematopoietic hyperplasia is a rare condition characterized by a solitary osteolytic and expansive bony lesion of the rib with a maximum size of 9 cm. Diagnosis is confirmed by a combination of clinical, radiologic and histologic features [6].

Lesions were radiolucent and had a thin and intact cortex. ill-defined linear areas of higher density or calcification were prominent and no soft tissue extension was seen. All previously reported cases had similar radiologic characteristics [6].

From the aspect of histopathology, FHH is a mix of hypercellular and fatty marrow. The cellularity was increased by approximately 50–95 %. In all of the previously reported cases, the marrow contained all hematopoietic cell lines (erythroid, myeloid and megakaryocytic cells) and no morphologically abnormal cells or metastatic carcinoma cells were seen. Bony tissue became osteoporotic at some parts (due to disruption and thinning of some of the bony trabeculae) and denser at other parts (due to thickening and distortion of trabeculae) [6].

There were only seven cases of FHH reported earlier in English literature.

In 1984 Gerald Eldelstein and Kyriakos presented first two cases of focal hyperplasia of the hematopoietic marrow causing rib lesions without any associated hematologic abnormality. Their first case was a 66-year-old black woman presented to ER with sudden onset vomiting, diarrhea, and abdominal discomfort. During medical evaluations, they accidentally found a 4.0 × 3.0 cm well delineated ovoid, expansible, and Lucent lesion arising near the ribs posterior articulation at a detailed roentgenogram. The second case was found during medical evaluations performed before surgical repair of inguinal hernia in a 71-year-old white man. None of their cases had any history of other benign or malignant tumors. Both patients underwent surgical excision of bony masses after symptomatic therapy. They concluded that these lesions are benign and are a form of pseudotumor, designated as "focal hematopoietic hyperplasia of rib" or "hematopoietic pseudotumor" [1].

The third case was a 46-year-old with A large, lytic mass on the posterior aspect of the sixth rib, reported in 1997 by Lorenzo M. Galindo. Despite the superficial location and significant size, the lesion was asymptomatic and was found during radiologic evaluations performed for assessment of diverticulitis. The radiologic studies suggested the presence of a neoplastic lesion; This lesion was fairly radiolucent and had trabeculated or linear areas; similar to radiologic characteristics of the previous two cases. The diagnosis was confirmed by fine needle aspiration biopsy (FNAB) [3].

The fourth case was a 24-year-old woman who presented with a rib mass incidentally found at chest radiograph while assessing a degenerative thyroid cyst. They performed computed tomography which revealed an expanding bony lesion with internal calcification arising from the head of the posterior rib and involved the transverse process of the third thoracic vertebra. They did not mention any signs of cortical destruction, soft tissue involvement or periosteal reaction. unexpectedly, the histopathologic study revealed the focal fibrosis and osteosclerosis which are suggestive of myelofibrosis. But then, fibrosis was limited to the endosteal surface. Before that case, there was no report of myelofibrosis associated with an expanding lesion of bone.

A 34-year-old female who was presented with complaints of chronic pain at the region of the 6th to 8th left side ribs was the fifth case of FHH. It was the only symptomatic case of FHH. She had no history of trauma or family history of malignancies. They performed positron emission tomography (PET) bone scan that revealed a hot area in the posterior aspect of the left 7th rib. A 250 mm in length lesion was radically excised through a left postero-lateral thoracotomy of the left 7th rib. They evaluated a cross section of the thickened part of the rib; uniform thickening of the marrow cavity without any focal lesions was seen. Further histopathologic evaluations, revealed hyperplasia of all hematopoietic cell types in their mature form and their precursor cells; same as previous other cases of FHH.

The sixth case was a 24-year old, professional athlete with a complaint of back pain following trauma. There was no abnormal finding in clinical evaluations. For evaluating the source of pain, the bone scan was performed, which did not show increased radiotracer accumulation; differently, the whole-body PET performed and demonstrated high uptake of the radiotracer in the vertebral body of L3. Also, lumbar spine MRI showed the vertebral lesion. They took a biopsy of the L3 vertebral body lesion and sent species for further histological examination which was suggestive of a chronic myeloproliferative disease. Moreover, they found hyperplastic bone marrow and fatty marrow (cellularity was increased by approximately 50–95 %) consisting of normal hematopoietic cells of all three lineages. There was also severe osteoporotic tissue while hyperactivity of osteoclastic cells was not obvious. He achieved satisfactory outcome after surgical resection of the lesion.

The seventh case was a 26-year-old female with palpable rib mass. Chest Radiograph demonstrated an expanding bony lesion. Computed tomography scan showed a 5.0 × 4.5-cm mass in the body of second rib associated with internal linear calcification and thinning of the inner surface without cortical destruction. With the initial diagnosis of osteochondroma (which is also a benign lesion of ribs). immediately she underwent surgical resection and species were sent for further microscopic evaluations. Hypercellular bone marrow containing three lineages of hematopoietic cells and their precursor with fatty marrow in the inter-trabecular spaces was observed. There was no evidence suggesting malignant conditions.

Based on our review of the literature, our case is the eighth reported case of FHH. None of the patients gave a history of trauma to the site involved or family history of malignancy. Only one of the previous cases had a history of trauma which was not at the definite site of the lesion. There was no association with hematological disorders or other malignancies that might have initiated FHH. Except for one case, others were found incidentally during radiologic studies for other non-relevant reasons. All cases share similar clinical, radiological and histological findings. Specific radiologic features made the chest routine radiographs and CT-scan, the best modalities for the diagnosis of FHH. Radiologically, these lesions are expansive and radiolucent masses with the thin cortex and containing areas of increased density or calcified trabeculae. Surrounding soft tissue involvement was not found in any of cases. Microscopic evidence in all reported cases including ours showed the characteristic pattern of mixed areas of hypercellular marrow and fatty marrow. There was no report of morphologically abnormal hematopoietic cell or malignant tumor cells. All patient underwent surgical resection, satisfactory results were achieved and no post-operative complication was reported. None of the cases experienced any episode of recurrence and there was no need for chemotherapy or re-surgery.

Based on our primary evaluation, the lesion was not thought to be malignant. NO malignant cell was reported at pathologic evaluations of fine-needle biopsy. However, it was probable that the specimen was obtained from the peripheral tissue and could not accurately rule out malignancy. In addition, our patient was suffering from severe localized pain. Thus, similar to previous cases, we decided to surgically remove the lesion to first relieve the pain and then confirm the diagnosis by histopathologic studies. Considering the possible harms following a total corpectomy especially in a young patient, we performed partial T5 and T6 corpectomy. During the operation, we completely resected the lesion and a safe margin which was determined the aid of rapid analysis of the frozen section to avoid local recurrence. Also the reactive tissue of cortex was excised for safety.

In discussing the differential diagnosis of FHH we should consider all benign or malignant lesions. Radiologic findings suggest some differential diagnosis for FHH including fibrous dysplasia, aneurysmal bone cyst, and The most complicated of all, plasmacytoma. From the aspect of histopathologic evaluations, chondrosarcoma and some other bony lesions with the manifestation of hematopoietic hyperplasia must also be considered among differential diagnosis. however, Myelolipoma and some hematological disorders including Thalassemia, chronic anemia, leukemia and myelofibrosis also present with increased hematopoiesis, hyperplastic and fatty marrow and thin bony trabeculae. Tumor-like hyperplasia of fat tissue and bone marrow, known as myelolipoma; are most frequently found in the adrenal gland [6,4].

Myelofibrosis is differentiated with concurrent hyperplasia of three hematopoietic cell lines which does not match with our findings.

In cases of Thalassemia evidence of bone involvement include widened marrow cavity, cortical thinning and marrow hyperplasia due to severe hemolysis. Though the bony involvement of Thalassemia is distinct from others with the medullary expansion of multiple bones (such as craniofacial bones, vertebrae, pelvic bones and ribs) and frequently involves skull. The posterior aspect of ribs shows cortical expansion. The patient suffers from anemia and is expected to have clinical features of anemia, skeletal deformity, and hepatosplenomegaly.

FHH is not associated with anemia and hepatosplenomegaly and none of the above mentioned clinical features were present in our patient [6,2,4].

Three main possible mechanisms explain the pathogenesis of FHH. First of all, these lesions may grow either due to a reactive process (secondary to trauma or inflammation) or developmental anomalies. None of the previously reported cases, including ours, reported any recognized trauma or history of inflammation prior to the diagnosis of FHH. Also, no primary lesions that may have led to these lesions were found in any of cases. On the other hand, most cases were diagnosed at an advanced age and it is against the theory of originating from developmental anomalies. Thus, we believe in young healthy patients, similar to our discussed patient, FHH can develop due to late and reactive change of either inflammation or some unrecognized benign tumor. However, it is not possible to determine the exact etiology, especially in a young healthy patient like our case [6,4].

Furthermore, it is possible that stress itself should be considered as a risk factor and can lead to the reconversion of yellow marrow to hematopoietic marrow [5].

Besides above-mentioned mechanisms, Stabler et al. demonstrated that hematopoietic hyperplasia can also be associated with malignancies and chronic anemia. Also, the granulocyte-colony-stimulating (GCS) factors that are used as an adjunct to radiation or chemotherapy treatments to decrease can lead to hyperplasia of the hematopoietic marrow [5].

The ethical committee of our institute gave us permission for current publication.

The patient gave informed consent and is aware of publication of this case report.

## 4 Conclusion

It is important to consider FHH of the rib among the differential diagnosis of solitary expanding bony lesions of ribs and vertebrae, especially when FNAB or small tissue biopsy specimens yield apparently normal marrow tissue and microscopic evaluation reveal mixed hyperplasia of marrow and fatty tissue.

## Funding

We had no source of funding

## Ethical approval

The ethical committee of neurosurgery research centre at Iran University of Medical Sciences is aware of current publication and gave permission.

## Consent

Our patient gave us informed consent after making sure her information are published anonymously

## Author contribution

- Reza Mollahoseini – Diagnosis and surgery – supervising this report.
- Paniz Motaghi – Review of articles and writing manuscript.
- Alireza Dastmalchi _ surgery and help in writing manuscript.
- Hanieh Zahm – Histopathologic evaluation.

## Registration of research studies

This study is not a clinical trial, thus has not been registered.

## Guarantor

We had no Guarantor except Dr. Reza Mollahosseini the first author.

## Provenance and peer review

Not commissioned, externally peer-reviewed.

## Declaration of Competing Interest

This article has no conflict of interest.
